# Cytotoxic Effect of a Novel Synthesized Carbazole Compound on A549 Lung Cancer Cell Line

**DOI:** 10.1371/journal.pone.0129874

**Published:** 2015-07-02

**Authors:** Refilwe P. Molatlhegi, Alisa Phulukdaree, Krishnan Anand, Robert M. Gengan, Charlette Tiloke, Anil A. Chuturgoon

**Affiliations:** 1 Discipline of Medical Biochemistry, School of Laboratory Medicine and Medical Sciences, University of KwaZulu-Natal, Durban, RSA; 2 Department of Physiology, School of Medicine, University of Pretoria, Pretoria, RSA; 3 Department of Chemistry, Faculty of Applied Sciences, Durban University of Technology, Durban, RSA; Winship Cancer Institute of Emory University, UNITED STATES

## Abstract

Increased death rates due to lung cancer have necessitated the search for potential novel anticancer compounds such as carbazole derivatives. Carbazoles are aromatic heterocyclic compounds with anticancer, antibacterial and anti-inflammatory activity. The study investigated the ability of the novel carbazole compound (Z)-4-[9-ethyl-9aH-carbazol-3-yl) amino] pent-3-en-2-one (ECAP) to induce cytotoxicity of lung cancer cells and its mechanism of action. ECAP was synthesized as a yellow powder with melting point of 240-247 °C. The 3-(4,5-dimethythiazol-2-yl)-2,5-diphenyl tetrazolium bromide (MTT), lipid peroxidation and comet assays were used to assess the cytotoxic effect of the compound on A549 lung cancer cells. Protein expression was determined using western blots, apoptosis was measured by luminometry (caspase-3/7, -8 and -9) assay and flow cytometry was used to measure phosphatidylserine (PS) externalisation. ECAP induced a p53 mediated apoptosis of lung cancer cells due to a significant reduction in the expression of antioxidant defence proteins (Nrf2 and SOD), Hsp70 (p < 0.02) and Bcl-2 (p < 0.0006), thereby up-regulating reactive oxygen species (ROS) production. This resulted in DNA damage (p < 0.0001), up-regulation of Bax expression and caspase activity and induction of apoptosis in lung cancer cells. The results show the anticancer potential of ECAP on lung cancer.

## Background

Cancer has become a second leading life-threatening disease accounting for high mortality rates after cardiovascular diseases worldwide [1, 2]. According to Globocan, in 2012 there were about 14.1 million new cancer cases and 8.2 million cancer related deaths globally, compared to the reported 12.7 million and 7.6 million in 2008. Majority of global cancer cases is reported in developing countries, with a 63% mortality rate [3]. An increase in lung cancer cases in developing countries including South Africa (SA) is associated with infectious diseases such as Acquired Immunodeficiency Syndromes (AIDS) and tuberculosis (TB) [2]. During 2006 alone about 4,525 lung cancer related deaths were reported in SA. An increase in lung cancer cases in SA is also attributed to other factors such as smoking, environmental pollutants, occupational exposure and changing lifestyles [2, 4].

Reactive oxygen species (ROS) are one of the major causes of malignancy. They are products of oxygen metabolism in cells and their functions range from cell signalling, homeostasis and antimicrobial effects [5]. Mammalian cells are continually exposed to ROS generated both extrinsically and intrinsically [6]. Chronic and persistent exposure to high ROS levels induces DNA, protein and lipid damage, and can result in induction of diseases such as cancer [7, 8]. In order to maintain tissue homeostasis in multicellular organisms, cell proliferation and death are tightly regulated by cell cycle and apoptosis processes. However, mutation of tumour suppressor gene(s) (e.g. p53) and elevated levels of ROS may allow for unregulated growth leading to cancer development. Mammalian cells contain defence systems to regulate oxidative damage, and these include proteins such as superoxide dismutase (SOD), nuclear factor erythroid 2-related factor 2 (Nrf2) and heat shock protein 70 (Hsp70) [9–12].

The survival rates for lung cancer patients undergoing surgery is poor, therefore there is need for more potent anti-lung cancer drugs. The challenge still is the ability to develop highly effective drugs specific for lung cancer with little or no side effects on normal cells [13]. Heterocycles have become an important class of organic compounds for research because of their numerous medical and agricultural applications [14].

Carbazoles are aromatic heterocyclic organic compounds, with a tricyclic structure comprising of two benzene rings each fused onto a five-membered nitrogen-containing ring [14, 15]. These synthetic or natural products function through interaction with the DNA; they damage DNA resulting in inhibition of synthesis of new DNA or RNA [16]. Carbazoles and their derivatives inhibit cancer growth by intercalating into DNA, inhibiting DNA topoisomerase II activity, as well as through formation of covalent DNA adducts mediated through oxidation by cytochrome P_450_ and peroxides [15]. Different natural and synthetic carbazole derivatives including ellipticine, olivacine, elliptinium acetate, mahanimbine, mukonine, koenoline and rebaccamycin have been reported to have antineoplastic activity [15].

Based on their reported antitumor, antibacterial and anti-inflammatory activities, different synthetic carbazole analogs have been synthesized from naturally occurring carbazoles [14, 15, 17]. Carbazoles and their derivatives are increasingly being targeted for potential use in cancer treatment owing it to their large π-conjugated system which, make it easy to introduce different functional groups into the rigid carbazole ring [15].

The study investigated the anticancer properties of ECAP on lung cancer cells and determined the anti-neoplastic pathway it modulates together with the associated proteins. It was hypothesized that ECAP induced apoptosis of A549 lung cancer cells through induction of oxidative stress.

## Materials and Methods

Carbazole derivative (ECAP) was synthesized at the Department of Chemistry (Durban University of Technology, SA). Peripheral blood mononuclear cells (PBMCs) were isolated from whole blood from a healthy male volunteer after obtaining Institutional ethical approval (BF 170/11). A549 cells were obtained from Highveld Biologicals (Johannesburg, SA), Cell culture reagents from Whitehead Scientific (Johannesburg, SA), Ellipticine (Sigma, St Louis, MO, USA) and Western blot reagents from Bio-Rad (USA). Other reagents were obtained from Merck (SA).

### Synthesis of (Z)-4-((9-ethyl-9H-carbazol-3-yl) amino)pent-3-en-2-one

A mixture of acetylacetone (1.02 mL, 0.01 mol), 3-amino-9-ethylcarbazole (2.1 g, 0.01 mol) and indium chloride (0.22 g, 0.001 mol) in ethanol (25 mL) was heated under reflux for 5 h. After completion of the reaction, the excess of solvent was evaporated. The residue was dissolved in ice/water and extracted with ethyl acetate. Combined organic layers were dried over anhydrous sodium sulphate. It was then purified on a silica gel column (eluent—petroleum ether: ethyl acetate (90:10)). The pure product was recrystallized from methanol.

**Fig 1:**
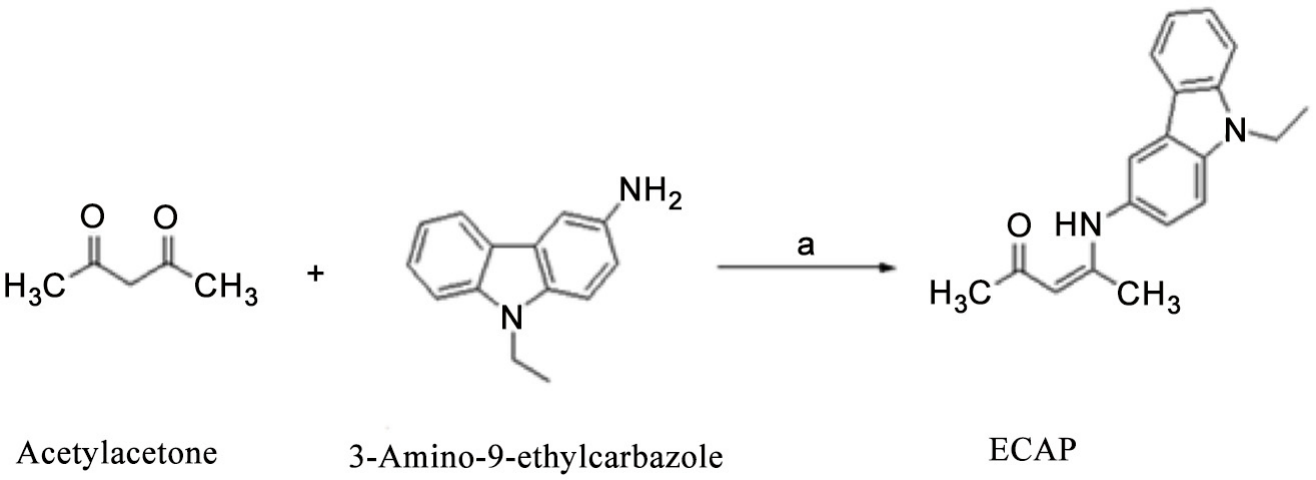
Schematic preparation of carbazole ECAP in Cl_3_/Ethanol, reflux, 5 h RT.

The title compound (Z)-4-((9-ethyl-9H-carbazol-3-yl) amino) pent-3-en-2-one (ECAP) was prepared in good yield by two-component reaction under indium chloride as promoter structure of the prepared compound (Fig 1). The structure of the prepared compound was then studied and characterized using Infrared (IR), Hydrogen-1 (^1^H) and Carbon-13 (^13^C NMR) spectroscopic techniques.

### Extraction of peripheral blood mononuclear cells

Peripheral blood mononuclear cells (PBMCs) were isolated from heparinised whole blood by density gradient centrifugation. Equal volumes of blood and Histopaque 1077 (Sigma, Germany) were aliquoted into 15ml conical tubes and centrifuged (400 g, 30 min). The buffy coat layer containing the PBMCs was aspirated and washed twice with 0.1M phosphate saline buffer (PBS; 400 g, 10 min).

### Tissue cell culture

A549 cells were cultured according to a previously described method [18]. In summary A549 cells were cultured at 37°C, 5% CO_2_ in Eagle’s minimum essential medium (EMEM) supplemented with 1% L-glutamine, 1% penicillin-streptomycin-fungizone and 10% foetal calf serum. Cell growth was monitored and cell culture medium (CCM) was changed as required. Confluent flasks were trypsinized and trypan blue was used for cell numeration. PBMCs obtained from a male donor were cultured at 37°C, 5% CO_2_ in Roswell Park Memorial Institute medium (RPMI) supplemented with 1% L-glutamine, 1% penicillin-streptomycin-fungizone and 10% fetal calf serum.

### Cell viability assay

Cell viability was conducted as previously described [18], with slight modification. The methyl thiazol tetrazolium (MTT) assay was used to determine the cytotoxicity of ECAP on PBMCs and A549 lung cancer cells. PBMCs and A549 cells (20, 000 cells/well) were incubated with different concentrations (0, 0.01, 0.05, 0.1, 0.25, 0.5, 0.6, 0.7 and 0.8 μg/ml) of ECAP and incubated in a 96-well microtitre plate in triplicate at 37°C, 5% CO_2_ for 24 h. Cells incubated with 1% DMSO were used as vehicle control (V control) and ellipticine at concentrations (0, 0.01, 0.025, 0.050, 0.10, 0.25, 0.6 and 0.8 μg/ml) was used as a positive control. After 24 h incubation, CCM/MTT salt solution (5 mg/ml) was added into each well and incubated at 37°C for 4 h. Supernatant was then removed and 100 μl/well dimethyl sulphoxide (DMSO) was added followed by incubation (1 h). The absorbance of the produced formazan was measured at 570 nm and reference wavelength of 690 nm using a Bio Tek μQuant spectrophotometer. GraphPad Prism V5.0 software was used to plot a concentration-response curve which was subsequently used to determine an IC_50_ value of ECAP on PBMCs and A549 cells.

### Lipid peroxidation assay for quantification of malondialdehyde (MDA)

Thiobarbituric acid assay (TBARS) was used to measure the ability of ECAP to generate ROS. The supernatant of the control, V control and ECAP treated cells was transferred into test tubes with 2% H_3_PO_4_ (200 μl), TBA/BHT solution (400 μl) and 7% H_3_PO_4_. A positive control of 1% MDA and a negative control of 3mM HCl (400 μl) was used. Sample pH was checked (pH 1.5) and heated at 100°C for 15 min. Samples were allowed to cool to room temperature (RT), followed by addition of 1.5 ml butanol, vortexed and then allowed to separate into distinct phases. The upper butanol phase was aliquoted into 96-well microtitre plate in triplicates. The optical density was measured at 532 nm with reference wavelength of 600 nm. The mean optical density was calculated for each sample and divided by the absorption coefficient of 156 mM^-1^ to obtain MDA concentration (μM) per treatment.

### DNA damage

The comet assay was used as previously described [18], with slight modification to determine DNA damage. The three treatments (control, V control and ECAP) were prepared respectively followed by removal of supernatant, and then trypsinized. Two slides per sample were prepared as follows; the first layer of 700 μl, 2% low melting point agarose (LMPA, 37°C), second layer of cells (25 μl), 1% LMPA (175 μl, 37°C) and gel red (1.5 μl), and the third layer of 1% LMPA (250 μl, 37°C), and then covered with microscope cover slides. After solidification, slides were submerged into cold lysing solution [2.5 M NaCl, 100 mM EDTA, 1% Triton X-100, 1 M Tris, 10% DMSO] and incubated at 4°C for 1 h. Following lysis, the slides were placed into electrophoresis buffer [300 mM NaOH, 1 mM Na_2_EDTA (pH 13)] for 20 min, and then electrophoresis was run at 25 V for 35 min. After electrophoresis, slides were washed three times for 5 min with neutralising buffer [0.4 M Tris (pH 7.4)]. Slides were then viewed with a fluorescent microscope (Olympus IXSI inverted microscope with 590 nm emission filters and 510–560 nm excitation). For each slide, 50 cells were captured and their comet tail lengths were measured in μm using Soft imaging system (Life Science-Olympus Soft Imaging Solution v5).

### Western blot analysis

The protein expression of p53, Bax, Bcl-2, Nrf2, SOD and Hsp70 in A549 cells was determined using western blot. Proteins were isolated from A549 cells using Cytobuster reagent supplemented with phosphate inhibitor (Roche, cat. no. 04906837001) and protease inhibitor (Roche, cat. no. 05892791001). Bicinchoninic acid assay (Sigma, Germany) was used for protein quantification. Proteins were standardized to a concentration of 3.966 mg/ml. Samples were then prepared in a Laemmli buffer, boiled for 5 min at 100°C and electrophoresed at 150 V for 1 h in 7.5% sodium dodecyl sulphate polyacrylamide (SDS-PAGE) using Bio-Rad compact power supply. Trans-Blot Turbo Transfer system was used to transfer the separated proteins onto nitrocellulose membranes at 20 V for 45 min. The 3% BSA in Tris-buffered saline [(TTBS)-NaCl, KCl, Tris, dH_2_O, Tween 20, pH 7.4)] containing 0.5% Tween20 was used to block membranes for 1 h, followed by an overnight incubation with primary antibodies [p53 (2521P), Nrf2 (8882), SOD (4266), Hsp70 (BD 610607) and Bcl-2 (3869); 1:5,000], 5 x wash with TTBS, 1 h incubation with secondary antibodies (anti-mouse: ab97046; 1:10,000) at RT and 5 x wash with TTBS (10 min each). β-actin (ab8226; 1:5,000) was used for protein normalization. A 50:50 (v/v) of Clarity Western luminal/enhancer solution and peroxide solution was added onto each electro-blotted nitrocellulose membrane to form the antigen-antibody complex. The generated signal was detected using the Alliance 2.7 image documentation system (UViTech). Protein expression was then analysed using UViBand Advanced Image Analysis software v12.14 (UViTech). The data was expressed as fold change (FC).

### Caspase-3/7, 8, 9 and ATP activities

Time dependent luminescence-based assays (6 h and 24 h) were used to assess the activities of caspase-Glo 3/7, 8, 9 and ATP. Following treatment with ECAP (6 h and 24 h), A549 cells were trypsinized, adjusted to 20,000 cells/well, followed by centrifugation at 3000g x (5 min, RT). Supernatant was removed; the cells were then re-suspended in 50 μl PBS/well for each treatment and then seeded into an opaque polystyrene 96-well microtitre plate in six replicates. The manufacturer’s guidelines were used to prepare Caspase-Glo 3/7, 8, 9 and ATP reagents (Promega). About 100 μl of reagent was added into specific wells, and then incubated in the dark (30 min, RT). The Modulus microplate luminometer was used for the subsequent measurement of the luminescence and the obtained data was expressed as RLU.

### Annexin-V-Fluos assay

Annexin stain assay was conducted as previously described [19], with slight modification. Phosphatidylserine (PS) translocation was determined using annexin-V-Fluos apoptosis detection kit (Roche). To each 100 μl A549 cell suspension, 100 μl of Annexin staining buffer and 100 μl of Annexin-V-Fluos labelling solution (annexin-V: propidium iodide: annexin staining buffer (1:1:50 vol/vol/vol)) were added in 1.5 ml tubes. The BD Accuri flow cytometer was used to capture data from stained cells. Cells were analysed using the Accuri C6 gating and CFlow Plus v1.0 analysis software. For each sample 50,000 events were analysed in triplicate. The results are presented as percentage of apoptosis where cells positive for Annexin-V (FITC) in the lower right quadrant (early apoptosis) and upper right quadrant (late apoptosis) were gated.

### Statistical analysis

GraphPad Prism v5.0 software (GraphPad Software Inc., La Jolla, USA) was used for statistical analysis. For all experiments, the obtained results of the triplicates were represented as means with standard deviation (SD). The statistical significance of the results was analysed with unpaired *t*-test and a 95% confidence interval. The values of *p < 0*.*05* were then considered statistically significant.

## Results

### Synthesis of (Z)-4-((9-ethyl-9H-carbazol-3-yl) amino) pent-3-en-2-one

**Fig 2:**
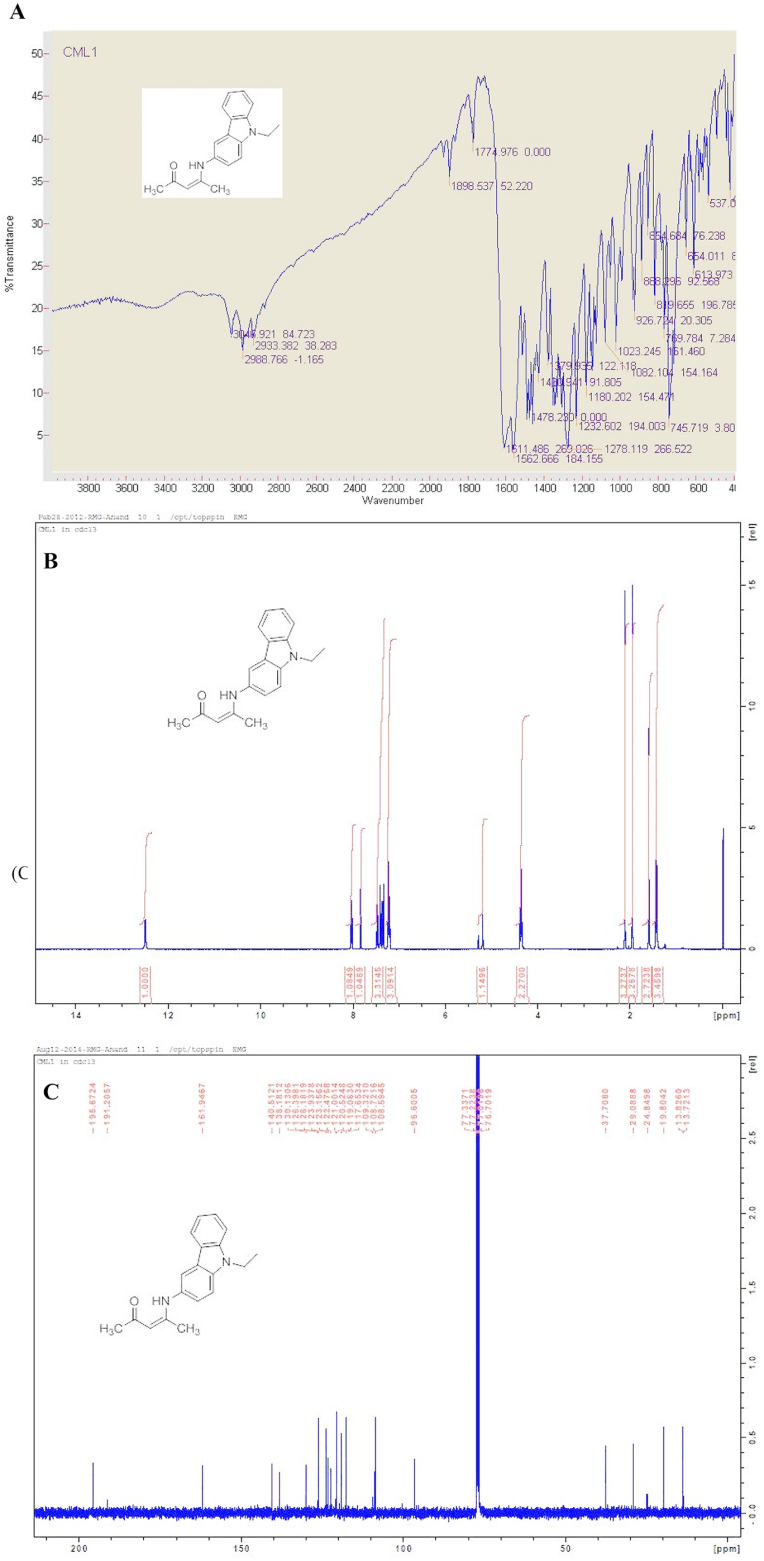
Characterization of the novel (Z)-4-((9-ethyl-9H-carbazol-3-yl) amino) pent-3-en-2-one (ECAP). (A) IR, (B) ^1^H-NMR and (C) ^13^C-NMR spectrums.

The resulting product was a yellow solid produced in 94% yield; melting point (mp): 240–247°C. Functional groups were predicted using IR (KBr, cm^-1^): 1774.97 (C = O), 3043.92 (N-H), 2988.76 (C-H) Alkanes, 1562.66 (C = C) Aromatic rings (Fig 2A). The ^1^H-NMR (400 MHz, CDCl_3_) was used to predict the ratio of the hydrogen number: δ (ppm) 12.59 (s, 1H), 8.20 (q, 1H), 7.8 (d, 1H), 7.45–7.40 (m, 2H), 7.35–7.30 (m, 3H), 5.3 (s, 1H), 4.35 (q, 2H), 2.25 (s, 3H) 1.95 (s, 3H), 1.45 (t, 3H) (Fig 2B). The ^13^C-NMR (400 MHz, CDCl_3_) was further used to predict the backbone of the molecule: δ (ppm) 195.67, 191.20, 161.94, 140.51, 138.18, 130.13, 126.39, 126.18, 123.93, 123.15, 122.47, 121.00, 120.52, 119.00, 117.65, 109.32, 108.72, 108.59, 96.60, 37.70, 29.08, 27.84, 19.80, 13.82, 13.72 (Fig 2C).

### Cell viability assay

**Fig 3:**
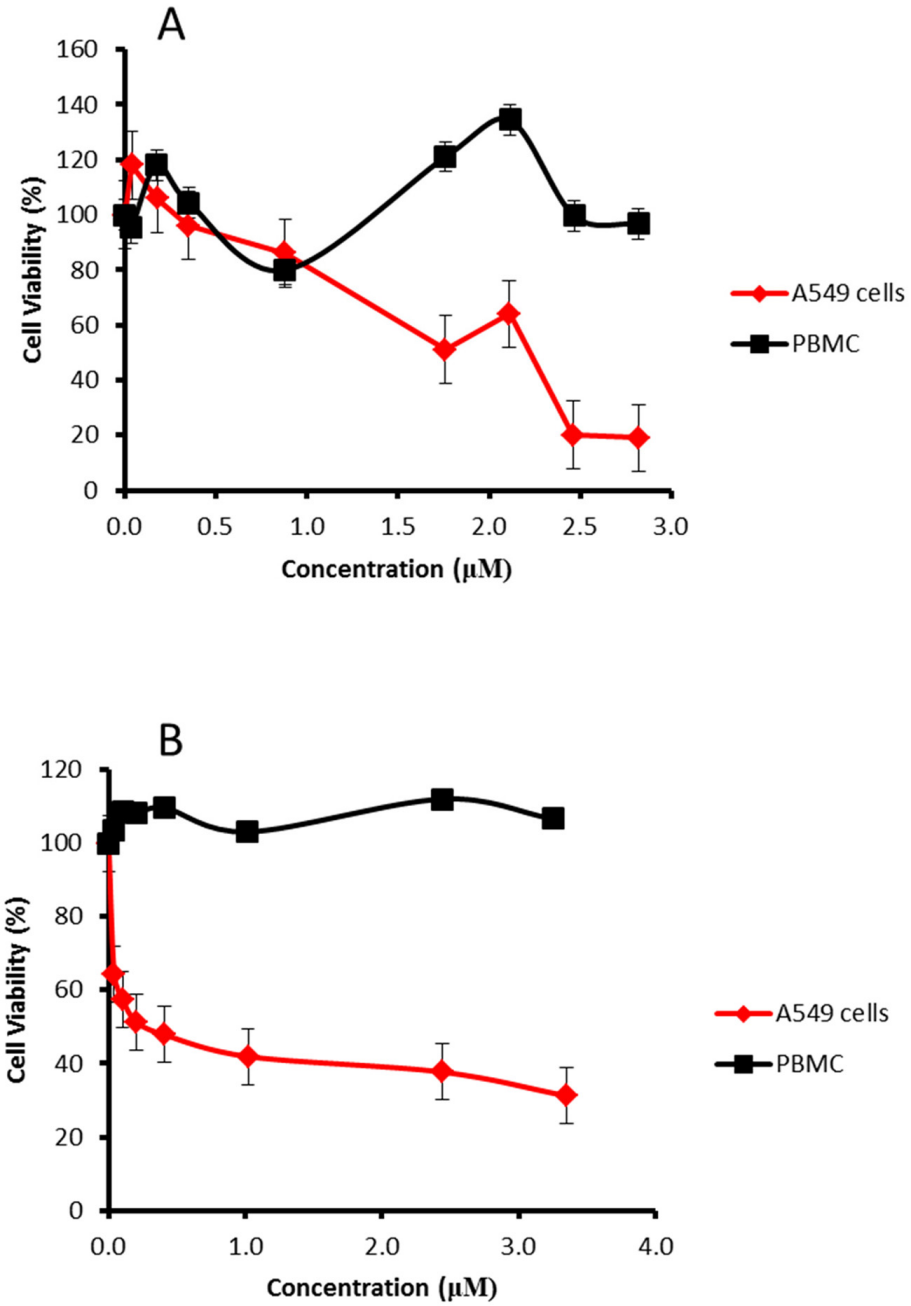
Dose-response curve showing the cytotoxic effect of ECAP and ellipticine on PBMCs and A549 lung cancer cell line. (A) The effect of (A) ECAP and (B) ellipticine on cell viability of PBMCs and A549 cells after 24 h treatment. [N = 3 replicates at 95% confidence interval].

The MTT assay was used to determine the cytotoxic effect of ECAP on normal healthy PBMCs and A549 lung cancer cells. Ellipticine (a potent neoplastic carbazole derivative) was used as a positive control (Fig 3), while 1% DMSO was used as a vehicle control (S1 Fig). Based on the vehicle control results (S1 Fig) and the previous DMSO cell viability results [20], 1% DMSO did not induce cytotoxicity, therefore ECAP was dissolved in 1% DMSO and used in all subsequent treatments. The cytotoxicity data represented a dose response relationship with respect to both ECAP and ellipticine (Fig 3).

Using the obtained dose-response curve, an IC_50_ value of 1.99 μM at 95% confidence interval was calculated for ECAP treated A549 lung cancer cells (Table 1) and subsequently used in all assays. Further, ECAP showed no cytotoxicity in normal healthy PBMCs (it was not possible to calculate IC_50_ value for PBMCs).

**Table 1:** IC_50_ values of ECAP and ellipticine on A549 lung cancer cell line after 24 h treatment.

A549 cells	
ECAP	1.99 μM
Ellipticine	0.32 μM

N = 3 replicates at 95% confidence interval

### Lipid peroxidation assay for quantification of malondialdehyde (MDA)

**Fig 4:**
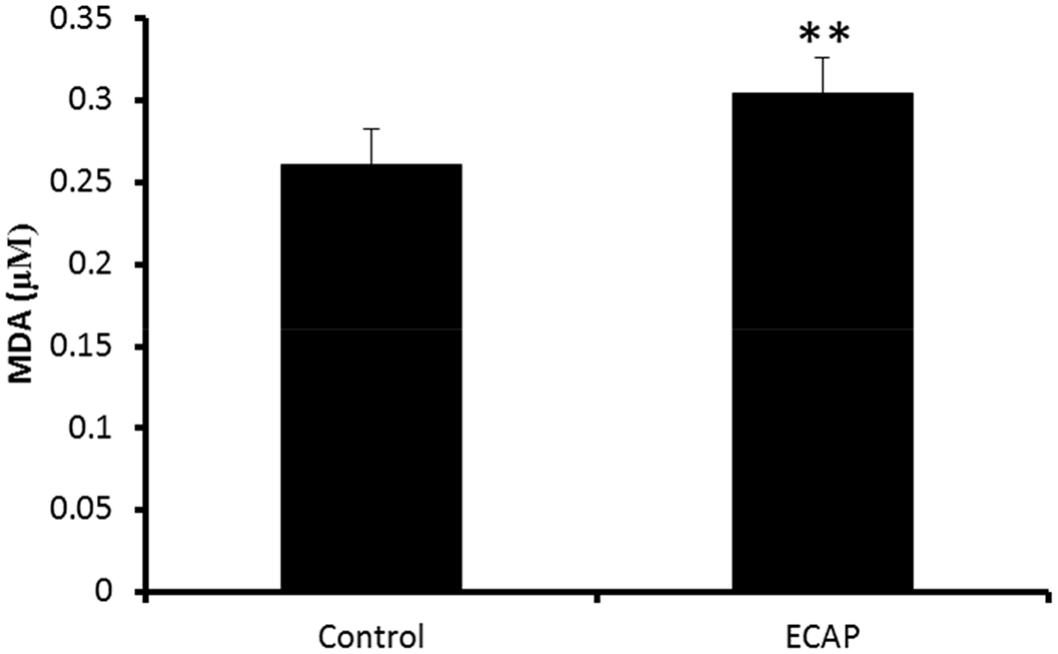
The effect of ECAP on lipid peroxidation on A549 lung cancer cells after 24 h incubation. (p < 0.0002) [*** significance compared to the control, number of replicates: n ≥ 3].

Lipid peroxidation, as measured by the MDA concentration, was significantly increased in ECAP treated cells compared to the control (0.305 ± 0.00490 μM vs 0.190 ± 0.007479 μM (control)) at 95% confident interval (Fig 4).

### DNA damage

**Fig 5:**
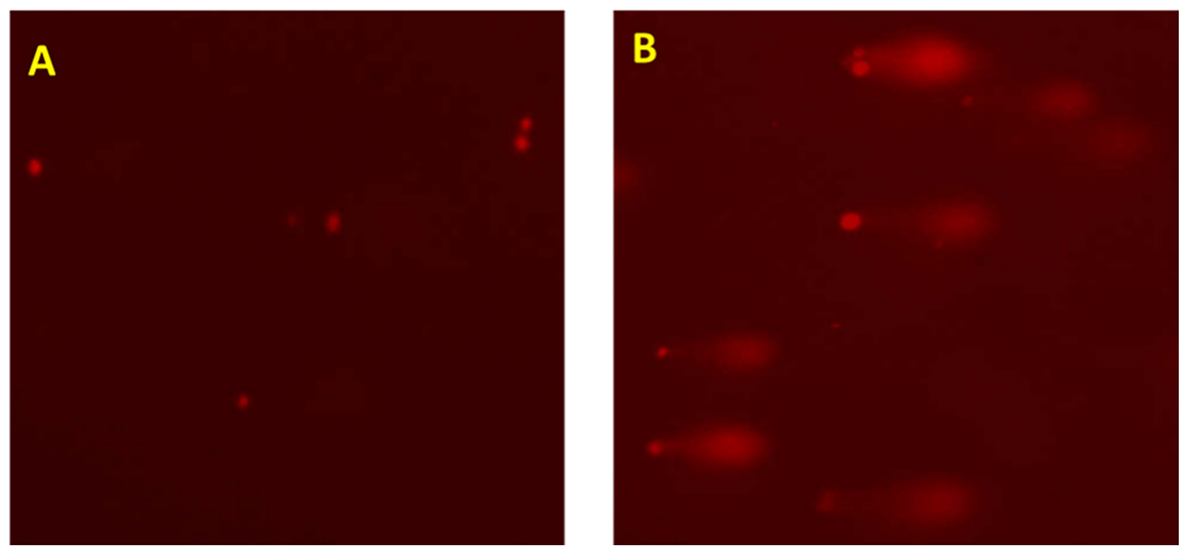
Comet assay showing DNA damage in A549 cells after 24 h treatment. (A) Control and (B) ECAP treated A549 cells. (p < 0.0001).

DNA damage was assessed using the comet assay. ECAP significantly increased the length of comet tails in treated cells compared to the control with 81.80 ± 1.19 μm vs 68.20 ± 1.61 μm (control) (Fig 5).

### Western blot analysis

**Fig 6:**
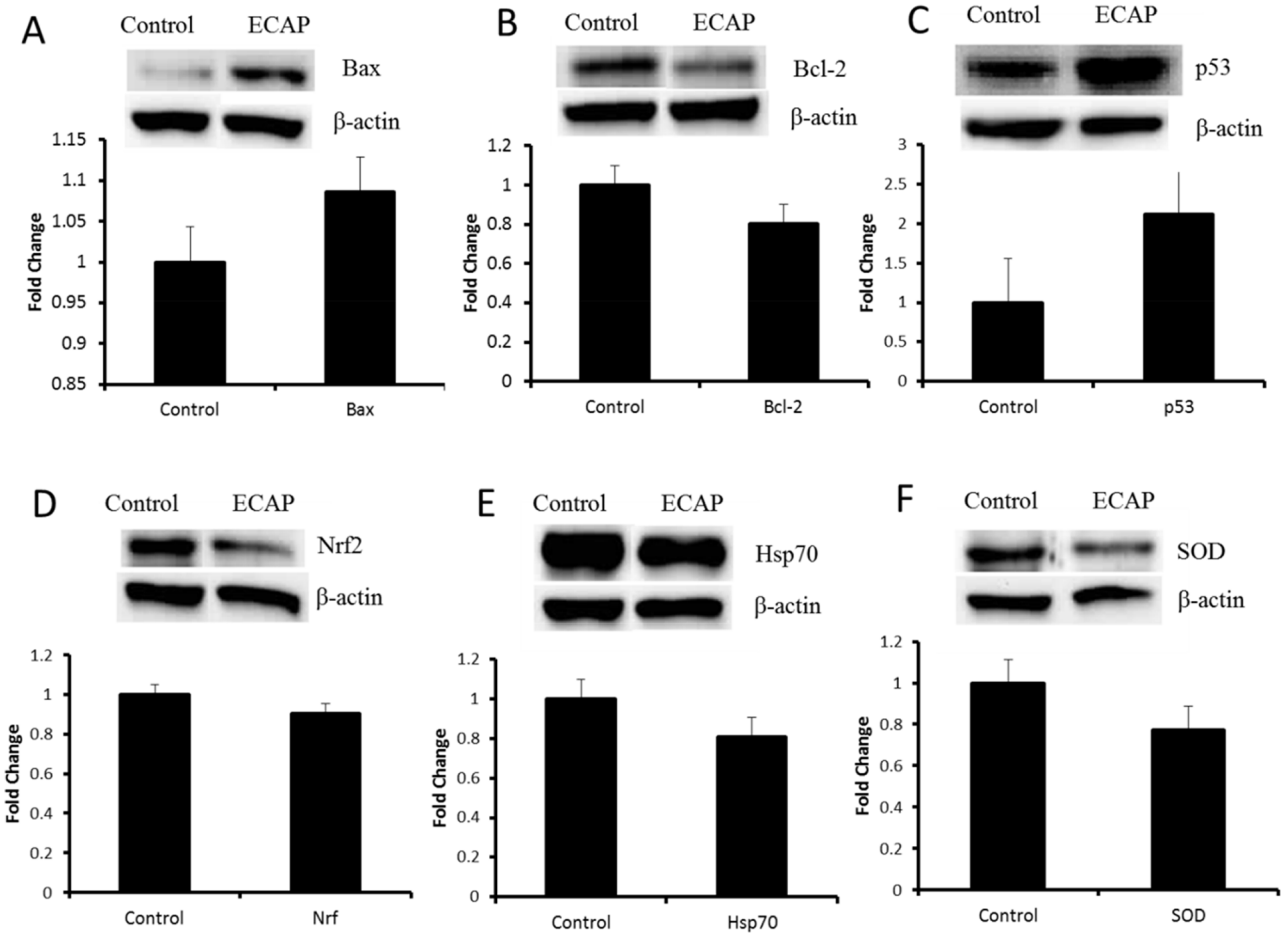
Western blot data shwing effect of the novel ECAP on protein expression in A549 lung cancer cell line. (A) Bax, (B) Bcl-2, (C) p53, (D) Nrf2, (E) Hsp70 and (F) SOD.

Western blotting was used to determine apoptotic proteins induced by ECAP in A549 lung cancer cells. Nrf2/Keap1 system regulates the expression of proteins such as SOD and heat shock proteins which are involved in the cellular antioxidant and anti-inflammatory defence [21]. A significant reduction in the expression of Nrf2 (p < 0.02*), Hsp70 (p < 0.02*) and SOD (p < 0.01**) was observed in ECAP treated cells compared to the control (Fig 6, S2 Fig). ECAP treated cell further demonstrated an increase in the expression of pro-apoptotic proteins such as p53 (p < 0.09) and Bax (p < 0.25) and a reduction in the expression of anti-apoptotic Bcl-2 (p < 0.0006***) (Fig 6, S2 Fig).

### Caspase-3/7, 8, 9 and ATP activities

**Fig 7:**
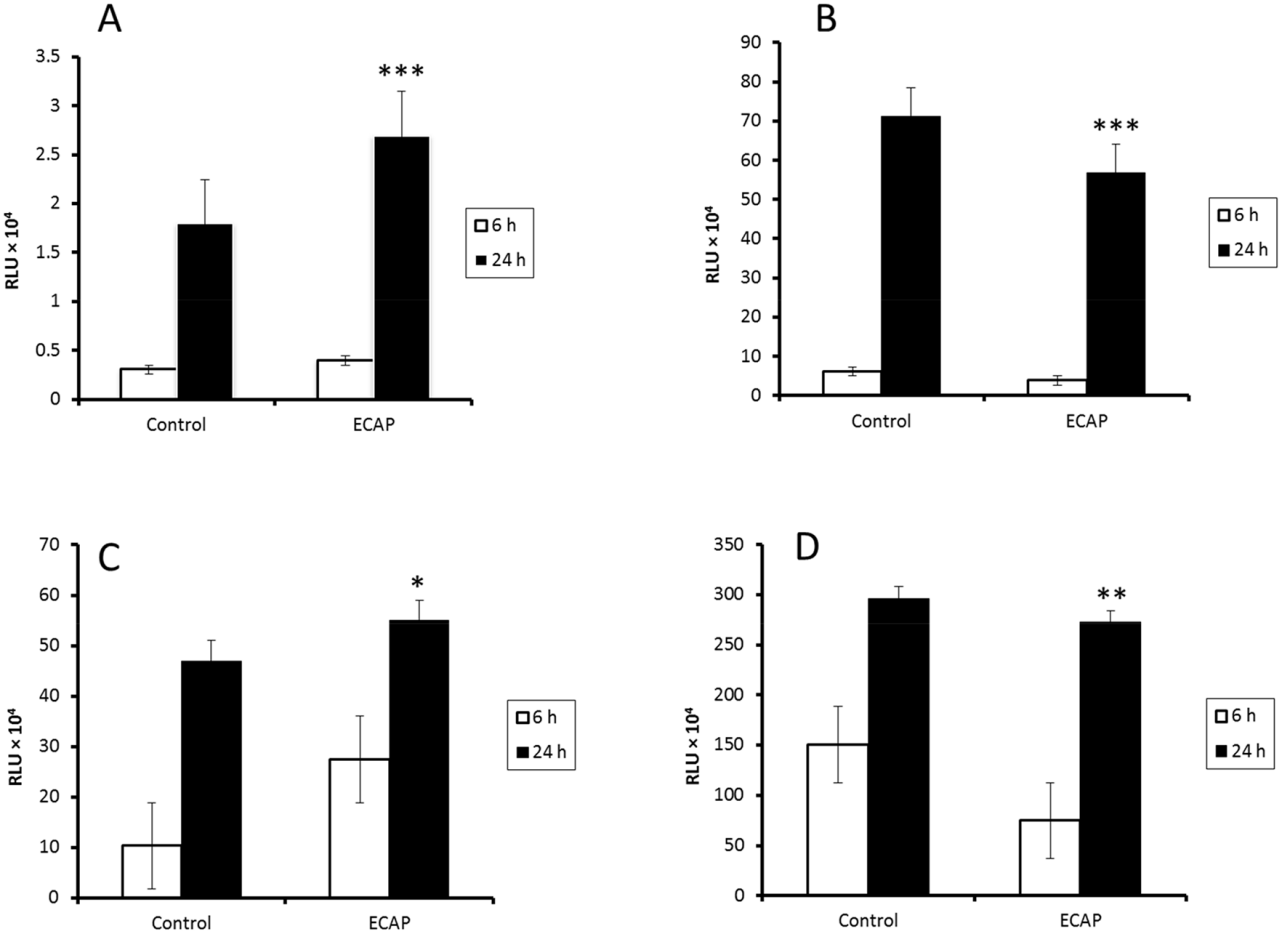
Time dependent effect of ECAP on caspase activity and ATP level. (A) The activity of Caspase-3/7 (6 h: p < 0.4036, 24 h: p < 0.0003), (B) Caspase-8 (6 h: p < 0.4364, 24 h: p < 0.0001), (C) Caspase-9 (6 h: 0.4171, 24 h: p < 0.0124) and (D) ATP (6 h: 0.4011, 24 h: 0.0011) levels in A549 lung cancer cell line after 6 h and 24 h incubation. [* denotes statistical significance with respect to the control and uncertainties represent standard deviation (SD) from the means. Number of replicates: n ≥ 3].

Caspases are ATP dependent enzymes that promote apoptosis. The time dependent effect of ECAP on the activity of caspases and ATP levels were measured in A549 lung cancer cells after 6 h and 24 h incubation (Fig 7).

### Annexin-V-Fluos assay

**Fig 8:**
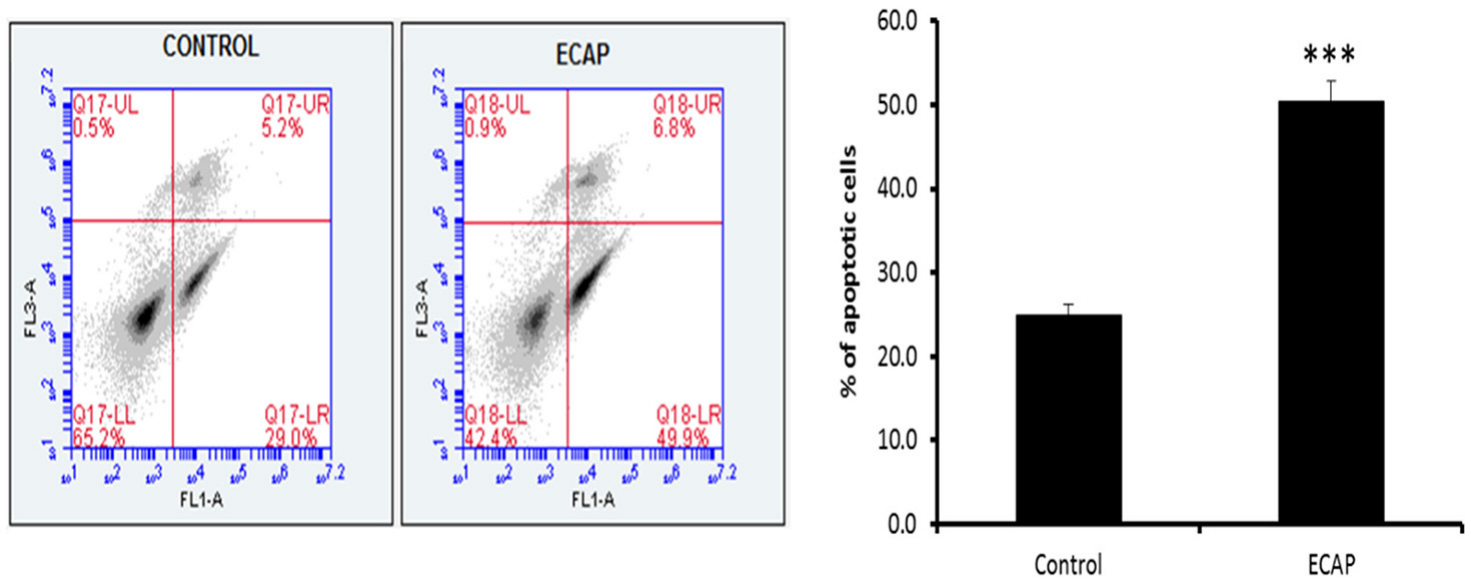
The effect of ECAP on the induction of apoptosis of A549 cells after 24 h treatment. [(p < 0.0001), *** significance compared to the control and number of replicates: n ≥ 3].

Translocation of the membrane phosphatidylserine (PS) is one of the markers of the early stages of apoptosis. After 24 h treatment, ECAP significantly induced apoptosis in A549 lung cancer cells (Fig 8, 50.4 ± 0.44%).

## Discussion

The cytotoxic activity of carbazoles and some of their analogs has been previously demonstrated on various cancer cell lines including lung, leukaemia and pancreatic cells [22–24]. The results of this study demonstrated the ability of ECAP to induce cytotoxicity on A549 lung cancer cells (Fig 3) and no cytotoxic effects on normal healthy human PBMCs (Fig 3). ECAP also demonstrated a wide range of apoptotic characteristics in A459 cells that included DNA fragmentation and PS translocation (Figs 5 and 8). In addition, ECAP induced changes in protein expression in A549 cells by reducing Nrf2, SOD, Hsp70 and Bcl-2 and overexpressing p53 and Bax (Fig 6). A time dependent increase in caspase activity and a decrease in ATP levels were also observed in the presence of ECAP. ECAP further induced elevation of lipid peroxidation in treated A549 lung cancer cells (Fig 4).

Anticancer therapies such as chemotherapy and radiotherapy make use of ROS overproduction to inhibit the proliferation of cancer cells [25]. Western blot data demonstrated a reduction in Nrf2 expression in ECAP treated A549 cells after 24 h incubation (Fig 6). Hence the observed reduction in the expression of Nrf2 may explain the reduced levels of expression of Hsp70 and SOD in A549 lung cancer cells (Fig 6). Down regulation of antioxidant defence systems is responsible for ROS elevation in cancer cells, as well as an induction of chronic oxidative damage on biomolecules such as DNA, proteins and lipids [18, 26]. ECAP significantly increased both lipid peroxidation in A549 lung cancer cells as measured by elevated MDA levels (Fig 4) and DNA damage (demonstrated by higher intensity and longer lengths of comet tails) (Fig 5).

The observed oxidative DNA damage in the comet assay could have resulted in the increased overexpression of serine 46 phosphorylated p53 in ECAP treated A549 lung cancer cells (Fig 6). It has been reported that DNA damage can activate p53 protein phosphorylation at serine 46 residue resulting in cell cycle arrest and subsequent DNA repair [27]. The western blot data further demonstrated Bax activation and reduction in the expression of Bcl-2 in the presence of ECAP (Fig 6). This suggests DNA damage was beyond repair, and increased p53 induced cell death through activation of pro-apoptotic proteins and reduction of anti-apoptotic proteins.

Bax activation induces mitochondrial depolarization thereby disrupting the electron transport chain (ETC) and affecting ATP levels. Roy and co-workers (2005) demonstrated mehanine, the carbazole alkaloid, to depolarize the mitochondrial membrane of U937 cells resulting in 60–40% reduction in cellular ATP levels compared to the control. In keeping with this observation, ECAP induced a significant 0.894 (24 h) fold and a 0.497 (6 h) fold decrease in cellular ATP levels in A549 lung cancer cells as compared to the control (Fig 7). Thus ECAP depolarized the mitochondrial membranes of A549 lung cancer cells resulting in cytochrome *c* release and the subsequent activation of caspase-9.

The cytotoxicity of ECAP in A549 lung cancer cells is associated with its ability to induce ROS overproduction, resulting in lipid peroxidation and DNA damage, and subsequent activation of increased expression of tumour suppressor proteins such as p53. In order to confirm the apoptotic action of this novel carbazole compound, luminometry was used to assess the effect of ECAP on the activity of the caspases. The activity of caspase-8 was 0.621 fold after 6 h and 0.797 fold after 24 h less in the presence of ECAP as compared to the controls (Fig 7). However, a time dependent increase in caspase-9 activity of 0.497 fold at 6 h and 1.17 fold at 24 h by ECAP in treated cells was observed as compared to the controls (Fig 7). Our data is in agreement with a previous study where mahanine was reported to induce apoptosis through a mitochondrial dependent pathway [16, 28]. ECAP induces death in A549 lung cancer cells through the intrinsic-mitochondrial pathway by activating caspase-9 (Fig 7). ECAP also resulted in increased activity of caspase-3/7 (1.31 fold change (6 h) and 1.50 fold change (24 h) as compared to the controls).

PS externalization, an early marker of apoptosis, was significantly higher in ECAP treated cells compared to the control, further demonstrating its potential anticancer properties. The data shows that the novel carbazole compound, ECAP, induces cytotoxicity and apoptosis in A549 lung cancer cells. ECAP induces apoptosis in cultured lung cancer cells by activating the caspase intrinsic mitochondrial-mediated apoptosis pathway; activated caspase-9 will activate the executioner caspases-3/7 and result in down-stream cleavage of proteins such as PARP.

## Conclusion

**Fig 9:**
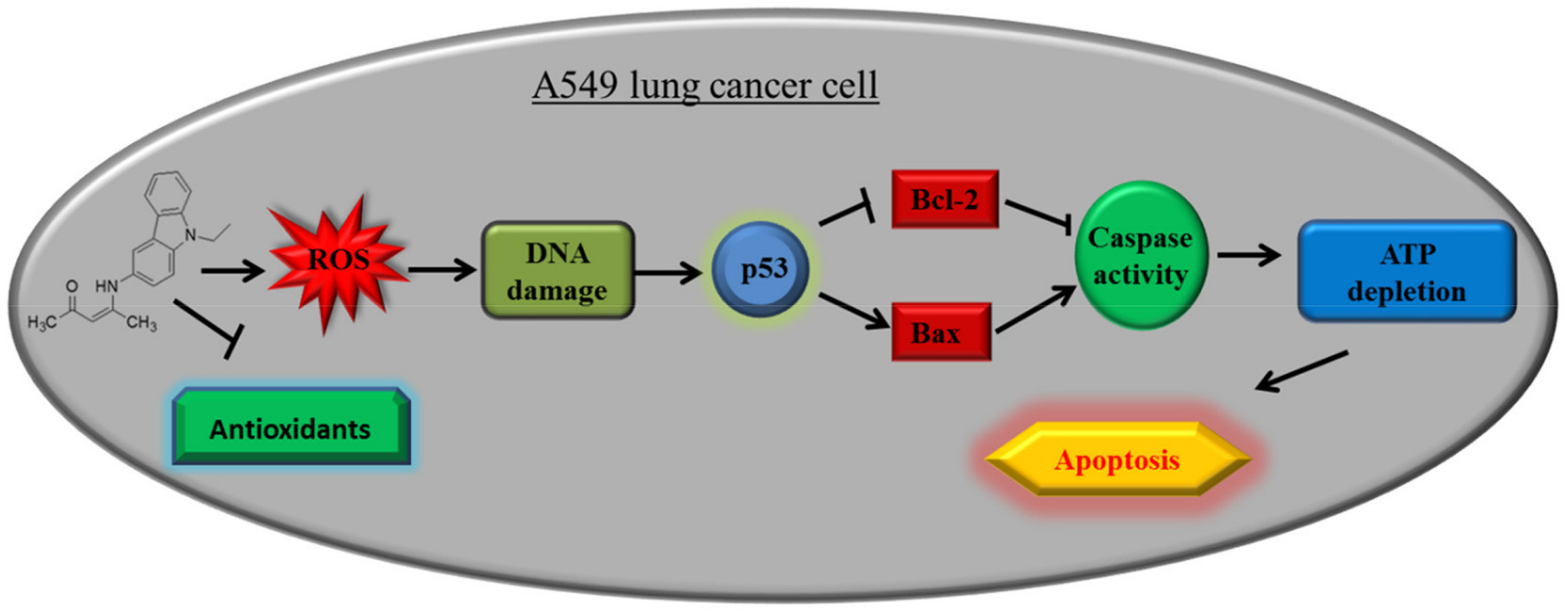
A schematic summary of apoptotic pathway of a novel carbazole compound (Z)-4-[9-ethyl-9aH-carbazol-3-yl) amino] pent-3-en-2-one on A549 lung cancer cells.

The study demonstrated the anticancer properties of ECAP to be due to its ability to induce p53 mediated apoptosis in lung cancer cells through activation of oxidative stress and reduction in expression of anti-apoptotic proteins and antioxidant defence proteins. This resulted in DNA damage and subsequent increased expression of pro-apoptotic proteins and apoptosis executioner molecules (Fig 9).

Our data shows that the novel carbazole compound (Z)-4-[9-ethyl-9aH-carbazol-3-yl) amino] pent-3-en-2-one possesses potential pharmaceutical properties as an alternative treatment for lung cancer. However, since this was an *in vitro* study, more studies need to be done to further characterize this novel compound and its mechanism of action in *in vivo* models. Future work includes monitoring the effect of methylation of ECAP on epigenetic changes and its effect on histones and microRNA expression. DNA relaxation assays will be used to evaluate its effect on topoisomerase activity in different cancer and non-cancer cell lines.

## Supporting Information

S1 Fig MTT assay measured in untreated (control) and 1% DMSO (vehicle control) treated A549 cells. The data showed no significant cytotoxicity.
(TIF)

S2 Fig Western blots showing the effect of ECAP on the expression of Bax, Bcl-2, p53, Nrf2, Hsp70 and SOD. The original western blots which were used for western blot analysis (Fig 6).
(TIF)
