# Supplementary figures and images for: Cytotoxic Effect of a Novel Synthesized Carbazole Compound on A549 Lung Cancer Cell Line
Source: PLoS One. 2015 Jul 2;10(7):e0129874. doi: 10.1371/journal.pone.0129874 (PMC4489925; doi:10.1371/journal.pone.0129874)

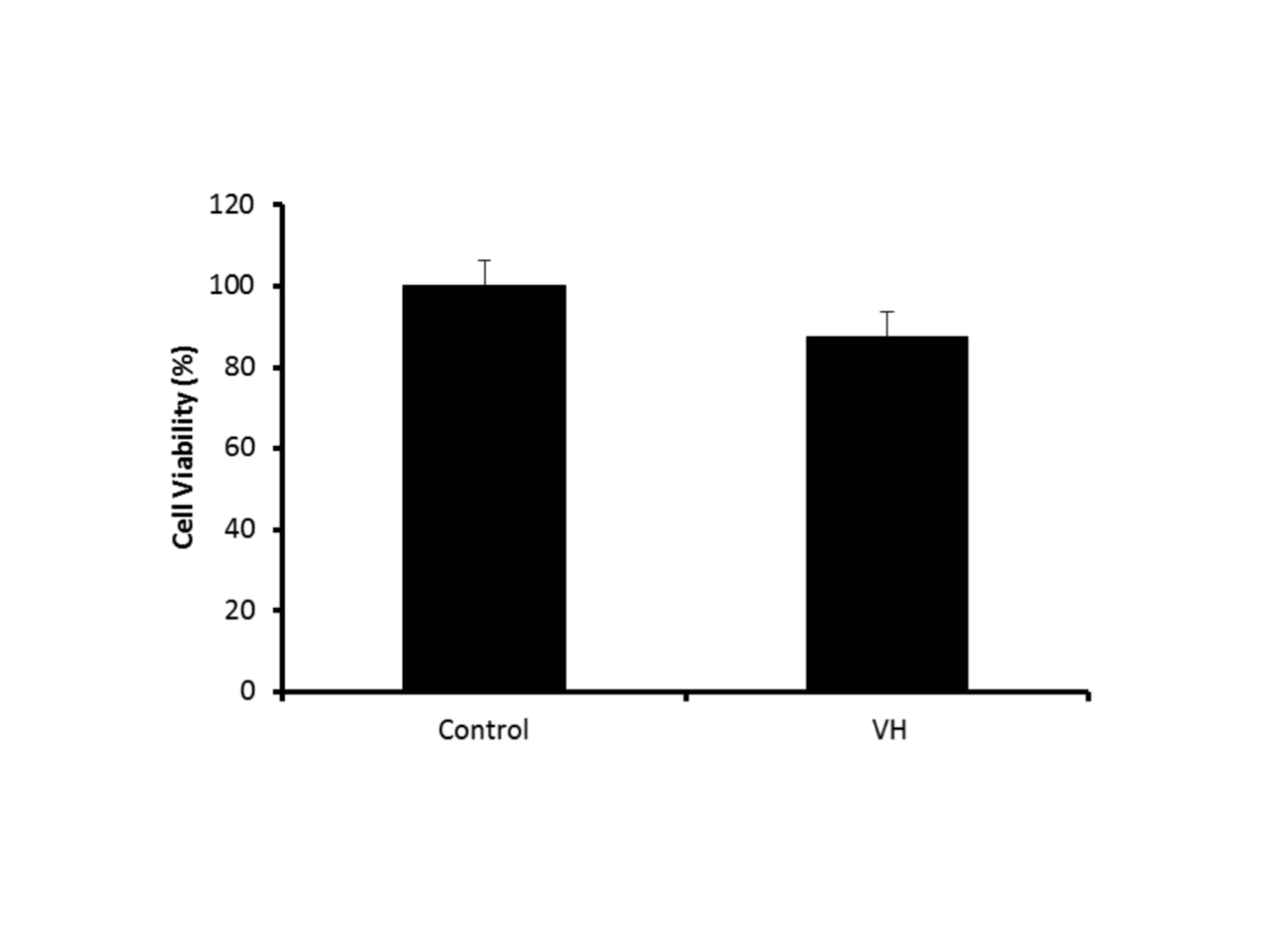

Supplement: S1 Fig — The data showed no significant cytotoxicity. (TIF) [file pone.0129874.s001.tif]

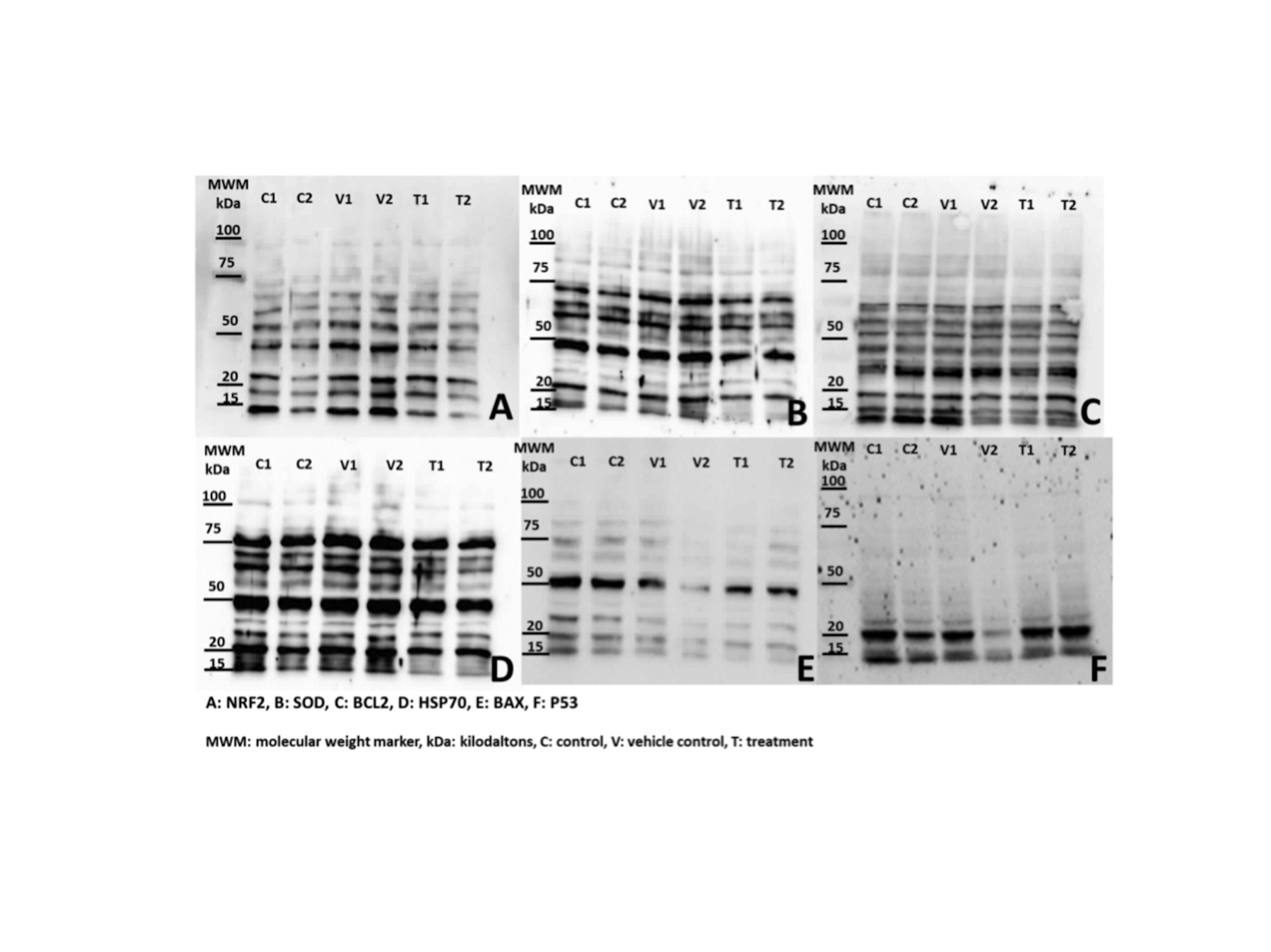

Supplement: S2 Fig — The original western blots which were used for western blot analysis (Fig 6). (TIF) [file pone.0129874.s002.tif]
